# Sparse-View CT Reconstruction Based on a Hybrid Domain Model with Multi-Level Wavelet Transform

**DOI:** 10.3390/s22093228

**Published:** 2022-04-22

**Authors:** Jielin Bai, Yitong Liu, Hongwen Yang

**Affiliations:** School of Information and Communication Engineering, Beijing University of Posts and Telecommunications, Haidian, Beijing 100876, China; bjl1027@bupt.edu.cn (J.B.); yanghong@bupt.edu.cn (H.Y.)

**Keywords:** CT reconstruction, sparsely sampled projections, hybrid domain method, directional and global artifact, multi-level wavelet transform

## Abstract

The reconstruction of sparsely sampled projection data will generate obvious streaking artifacts, resulting in image quality degradation and affecting medical diagnosis results. Wavelet transform can effectively decompose directional components of image, so the artifact features and edge details with high directionality can be better detected in the wavelet domain. Therefore, a hybrid domain method based on wavelet transform is proposed in this paper for the sparse-view CT reconstruction. The reconstruction model combines wavelet, spatial, and radon domains to restore the projection consistency and enhance image details. In addition, the global distribution of artifacts requires the network to have a large receptive field, so that a multi-level wavelet transform network (MWCNN) is applied to the hybrid domain model. Wavelet transform is used in the encoding part of the network to reduce the size of feature maps instead of pooling operation and inverse wavelet transform is deployed in the decoding part to recover image details. The proposed method can achieve PSNR of 41.049 dB and SSIM of 0.958 with 120 projections of three angular intervals, and obtain the highest values in this paper. Through the results of numerical analysis and reconstructed images, it shows that the hybrid domain method is superior to the single-domain methods. At the same time, the multi-level wavelet transform model is more suitable for CT reconstruction than the single-level wavelet transform.

## 1. Introduction

In computed tomography (CT) reconstruction, an exact reconstruction requires consecutive 180-degree parallel or fan beam scan data [1]. However, in order to follow the principle of “ALARA” (as low as reasonably achievable) [2] or because of the limited scanning conditions in the actual working environment, we often failed to obtain complete projection data, which leads to serious artifacts during reconstruction. Image reconstruction from incomplete projection data is essentially an ill-posed inverse problem that is difficult to converge to a correct solution and has become a challenging but hot topic in CT imaging. In this paper, we focus on the problem of reconstruction from sparsely sampled projection data that is sparse-view CT reconstruction.

Traditional analytic methods, such as filtered back-projection (FBP) algorithm [3], are highly dependent on data completeness, while iterative reconstruction algorithms do not require complete projections. However, classical iterative reconstruction algorithms, such as Algebraic Reconstruction Technique (ART) [4] and Simultaneous Algebraic Reconstruction Technique SART [5], still have problems such as geometric artifacts, high image noise, and time-consuming iteration steps. Compressed sensing (CS) [6] provides a new idea for incomplete projection reconstruction. With the guidance of CS theory, Sidky et al. [7] combined the SART algorithm with a total variation (TV) minimization method to solve the incomplete projection reconstruction problem, namely the SART-TV algorithm. The experiment showed that the TV-based method can reconstruct a high quality Shepp–Logan phantom using only 20 views of projection data. Moreover, a new iterative reconstruction algorithm based on TV regularization was proposed by Sidky et al., which is called adaptive steepest descent projection onto convex sets (ASD-POCS) [8].

In recent years, deep learning (DL) has been widely applied in computer version, such as image segmentation [9], denoising [10] and super-resolution reconstruction [11]. In 2016, Wang pointed out the application potential of deep learning in CT imaging [12]. With the further application of artificial intelligence in medical imaging, deep learning stands out in CT reconstruction, such as low-dose CT, limited-angle CT, and sparse-view CT [13,14,15,16,17]. FBPConvNet [13], residual encoder–decoder convolutional neural network (RED-CNN) [14], and Wavelet Domain Residual Network (WavResNet) [15] are typical networks for CT image denoising.

Based on the persistent homology analysis [18], which proved that the residual manifold of streaking artifacts is much simpler than the original one, Han et al. [19] proposed a residual network for directly learning the streaking artifacts in sparse-view CT. At the same time, the receptive field is enlarged by U-Net structure to capture the globally distributed streaking artifacts. Xie et al. [20] improved GoogLeNet [21] to remove the artifacts of sparse-view CT, which showed good reconstruction performance on 60 and 120 views. Zhang et al. [22] presented a neural network DD-Net for FBP reconstructed images, which utilized the advantages of DenseNet and deconvolution to suppress the artifacts caused by sparsely sampled projections. Lee et al. [23] used a multi-level wavelet convolutional neural network (MWCNN) [24] for CT restoration.

All the above DL algorithms are post-processing methods. However, when there are few projections, the artifacts in image domain are serious, which will cause the image details to be blurred, so it is not enough to eliminate the artifacts only in image domain. Effective information can be added to radon domain to reduce the generation of artifacts in the image domain caused by the partial absence of projections. Liang et al. [25] developed a residual network for sparse-view CT that predicts missing sinogram information. In essence, the completion of projection data can be regarded as an image repair problem, which restores the missing part by using the existing image information. Ghani et al. [26] used Conditional Generative Adversarial Network (CGAN) to learn sinogram repair, which showed great performance in sparsely sampled projections. Jin et al. [27] added a projection completion network based on context–encoder to their approach to generate the missing sinogram region influenced by its surroundings. The encoder captures the image context information and connects it to the decoder through the full connected layer to generate the missing sinogram features.

Although artifact elimination is an effective method for reconstructed CT, the inherent statistical properties of medical images are often ignored in image post-processing. However, the reconstructed image from the completed projection may cause secondary artifacts in the image domain due to slight inconsistencies. Hybrid-domain-based methods have been proposed to deal with the reconstruction problem at a deeper level. Yuan et al. [28] combined projection domain and image domain for sparse-view CT denoising. First, a super-resolution network was used to interpolate sinogram in the projection domain, and then the U-Net network was used in the image domain for image enhancement. Lee et al. [29] proposed a reconstruction method for sparsely sampled projections that applied a fully convolutional network and wavelet transform to a hybrid domain (combination of radon and image domains), and achieved a better reconstruction effect than that of any single domain. Hu et al. [30] also proposed a hybrid-domain neural network (HDNet), which is a two-stage method in which one stage only focuses on one domain. The HDNet can be further used in cone-beam imaging. Some studies not only combines image domain with a projection domain, but also extends the idea of domain combination to other domains, such as image gradient domain [31], frequency domain [32], and so on.

Inspired by the work of domain joint CT reconstruction, we develop a radon-image-joint domain reconstruction model based on projection data and spatial data wavelet transform. It consists of a radon domain projection completion module and an image domain detail refinement module. Moreover, MWCNN is used as the basic network for feature extraction in multi-scale wavelet domains, which has the powerful modeling ability for spatial context and inter-subband dependencies. It performs the enhancement of streaking artifacts detection and edge information restoration.

In summary, the key contributions of this paper include:

- On the basis of single domain restoration, a hybrid domain reconstruction model for sparse-view CT is proposed, which consists of a projection completion module in radon domain and an image restoration module in image domain. Wavelet domains of projection data and image data are embedded in two modules respectively to better extract spatial features and recover texture details. Moreover, the proposed model is end-to-end learning through the differentiable FBP operator.
- Multi-level wavelet packet decomposition is utilized to replace the pooling operator and enlarge the effective receptive field. Experimental results have shown that a multi-resolution network with a multi-level wavelet transform can effectively suppress globally distributed streaking artifacts.
- A deep residual learning framework is proposed to learn artifacts. Once the artifacts are estimated, an artifact-free image can be obtained by subtracting the estimated results.

## 2. Architecture

### 2.1. Wavelet Decomposition

It can be observed that the artifacts have two major characteristics: directionality and global distribution. It has been proved that learning in the wavelet domain is superior to that in the original image domain because the artifacts noise is directional, and the wavelet transform with directional filters can effectively decompose the directional component of noise [15]. Global distribution requires the network to have a large receptive field. One strategy is to use the fully convolution network (FCN), pooling the image after convolutions to reduce the size of the image and increase the receptive field at the same time. However, such a task is similar to image restoration, in which the input and output of the network have the same size, so the feature map with smaller size after pooling needs to be restored to the original image size for prediction through up-sampling. However, the pooling operation is not reversible. Once the average or maximum pooling is carried out, the new feature space cannot retain all the information of the original feature space. In order to reduce information loss, 2D discrete wavelet transform (2D-DWT) [33] is used to replace the pooling layer in this paper. Since DWT is reversible, such a downsampling scheme can preserve spatial information without loss.

**Figure 1:**
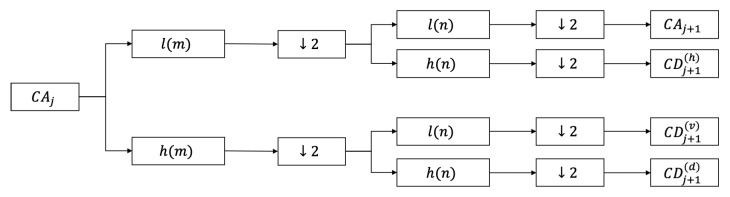
The procedure of 1-level 2D-DWT decomposition.

We use Haar [34] transform to implement 2D-DWT to extract multi-resolution features. As shown in Figure 1, for an M×N image, a low-pass filter *l*(*m*) and a high-pass filter *h*(*m*) are firstly used to convolve with each row of the two-dimensional image, respectively. Then, we discard the odd columns. Next, each column of the $\frac{M}{2}\times N$ array is convolved with *l*(*n*) and *h*(*n*) and the odd rows are discarded. After a 2D-DWT, we can obtain four sub-images $CA_{j+1}$, $CD_{j+1}^{\left(h\right)}$, $CD_{j+1}^{\left(v\right)}$ and $CD_{j+1}^{\left(d\right)}$. The low frequency component $CA_{j+1}$ can be further decomposed by the same method.

The inverse wavelet transform (IWT) can be used to accurately reconstruct the downsampled signal because of the biorthogonal characteristic of wavelet transform. The IWT is given by

(1) $$CA_{j}=IWT\left(CA_{j+1},CD_{j+1}^{\left(h\right)},CD_{j+1}^{\left(v\right)},CD_{j+1}^{\left(d\right)}\right).$$

**Figure 2:**
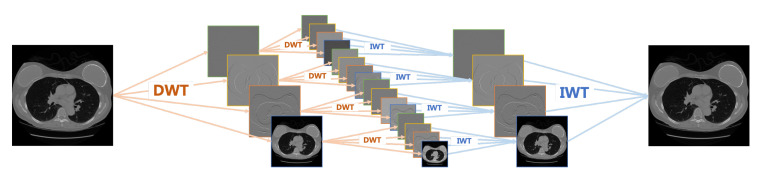
Multi-level WPT architecture.

Different from the wavelet transform, the wavelet packet transform (WPT) decomposes not only the low frequency component but also the high frequency components. Wavelet packet decomposition is a more widely used wavelet decomposition method, which is applied to signal decomposition, coding, denoising, compression, and so on. Figure 2 shows the process of image decomposition and reconstruction using multi-level WPT.

In fact, if a nonlinear mapping is added between any two layers of wavelet, the whole process can be regarded as a special case of FCN, that is, WPT is used to replace the original pooling layer. Due to the biorthogonal nature of WPT, i.e., reversibility, downsampling can be done without loss of information. In addition, compared with traditional convolutional neural network, the wavelet transform can capture the frequency and spatial information of the feature map simultaneously [35,36], which may help to preserve the detailed texture.

### 2.2. Reconstruction Model

#### 2.2.1. Deep Learning Network

**Figure 3:**
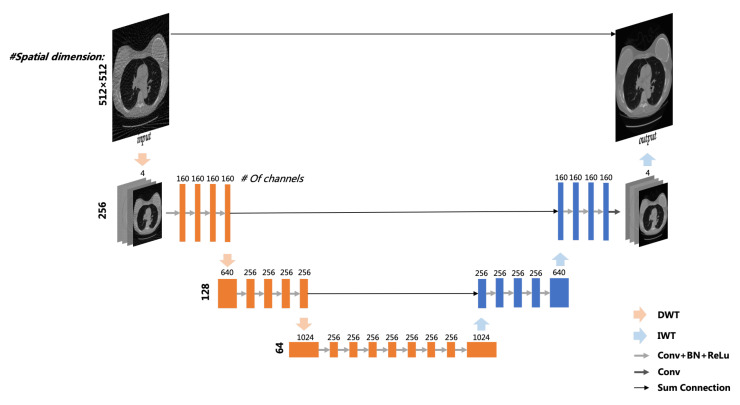
Multi-level wavelet CNN architecture.

According to the research in Section 2.1, we apply the multi-level wavelet convolutional neural network (MWCNN) to the hybrid domain reconstruction model. As shown in Figure 3, a CNN block composed of four convolution layers is added between any two layers of wavelet transform. In other words, all sub-band images decomposed by this layer of wavelet transform are the input of this CNN block, and a compact representation is learned as the input of the next layer of transform. Each layer of the block consists of the operations of convolution (Conv) with 3 × 3 filters, batch normalization (BN) and rectified linear unit (ReLU), except for the last layer of the last CNN block, which does not contain BN and ReLU operations.

MWCNN is based on U-Net [37] architecture, which consists of a contracting subnetwork and an expanding subnetwork. The network contains 30 layers, and more details of network settings can be referred to in Figure 3. In order to enrich the representation of features and reduce the amount of computation, the feature maps of the encoding network and the decoding network are combined by the method of sum by element, and the feature information of the encoder can be transmitted directly to the decoder.

In the CT reconstruction of incomplete projections, artifacts are similar even if the artifact-free images differ greatly. This means that it is easier to learn artifacts than the image without artifacts. Meanwhile, CT images have very high texture details, which are often difficult to estimate directly from reconstructed images. Therefore, artifacts in the reconstructed image can be directly learned and then an artifact-free CT image can be obtained by subtracting the estimated results. The reconstructed image with incomplete projections can be regarded as the superposition of the artifact-free image and artifacts. We train a mapping function of a reconstructed image (artifact image) to artifacts through MWCNN, and then use it to estimate the residual map.

L2 norm is used as the loss function of the single network. Specifically, it is assumed that it represents network parameters, and represents network output. Let ${\left\{(x_{i},y_{i})\right\}}_{i=1}^{N}$ be a training set, where $x_{i}$ represents the *i*-th input image and $y_{i}$ represents the artifact residual map of $x_{i}$ and the corresponding ground truth. The loss function is expressed as

(2) $$L\left(\theta \right)=\frac{1}{2N}\sum_{i=1}^{N}{∥F\left(x_{i},\theta \right)-y_{i}∥}_{2}^{2}.$$

#### 2.2.2. Relationship between WPT Operator and MWCNN

In MWCNN, the wavelet decomposition operation replaces the pooling layer for downsampling. More precisely, wavelet decomposition is performed on the feature map of each channel to avoid the loss of spatial features. As the network goes deeper, the level of decomposition increases, and the feature maps of different channels essentially represent different high-frequency and low-frequency subbands. It can be seen that MWCNN is a generalization of WPT, and degrades to a multi-level WPT process when the CNN block is only regarded as a mapping module.

In 2D Haar wavelet transform, there are four filters $f_{LL}$, $f_{LH}$, $f_{HL}$ and $f_{HH}$, and they are defined as

(3) $$f_{LL}=\left[\begin{array}{cc} 1 & 1\\ 1 & 1 \end{array}\right],f_{LH}=\left[\begin{array}{cc} -1 & -1\\ 1 & 1 \end{array}\right],f_{HL}=\left[\begin{array}{cc} -1 & 1\\ -1 & 1 \end{array}\right],f_{HH}=\left[\begin{array}{cc} 1 & -1\\ -1 & 1 \end{array}\right].$$

For an image x, the (i,j)-th pixel of subband image $x_{1}$ ($(f_{LL}⊗x)↓2$) can be expressed as $x_{1}\left(i,j\right)=x\left(2i-1,2j-1\right)+x\left(2i-1,2j\right)+x\left(2i,2j-1\right)+x\left(2i,2j\right)$. The expression of other subbands $x_{2}$ ($(f_{LH}⊗x)↓2$), $x_{3}$ ($(f_{HL}⊗x)↓2$), and $x_{4}$ ($(f_{HH}⊗x)↓2$) can be obtained analogously, and written as

(4) $$\begin{array}{c} x_{2}\left(i,j\right)=-x\left(2i-1,2j-1\right)-x\left(2i-1,2j\right)+x\left(2i,2j-1\right)+x\left(2i,2j\right),\\ x_{3}\left(i,j\right)=-x\left(2i-1,2j-1\right)+x\left(2i-1,2j\right)-x\left(2i,2j-1\right)+x\left(2i,2j\right),\\ x_{4}\left(i,j\right)=x\left(2i-1,2j-1\right)-x\left(2i-1,2j\right)-x\left(2i,2j-1\right)+x\left(2i,2j\right). \end{array}$$

Correspondingly, x can be obtained by

(5) $$\begin{array}{c} x\left(2i-1,2j-1\right)=(x_{1}\left(i,j\right)-x_{2}\left(i,j\right)-x_{3}\left(i,j\right)+x_{4}\left(i,j\right))/4,\\ x\left(2i-1,2j\right)=(x_{1}\left(i,j\right)-x_{2}\left(i,j\right)+x_{3}\left(i,j\right)-x_{4}\left(i,j\right))/4,\\ x\left(2i,2j-1\right)=(x_{1}\left(i,j\right)+x_{2}\left(i,j\right)-x_{3}\left(i,j\right)-x_{4}\left(i,j\right))/4,\\ x\left(2i,2j\right)=(x_{1}\left(i,j\right)+x_{2}\left(i,j\right)+x_{3}\left(i,j\right)+x_{4}\left(i,j\right))/4. \end{array}$$

According to the calculation method of Haar wavelet transform described by Equations (4) and (5), we can perform DWT and IWT on the feature maps in MWCNN. It is worth noting that the wavelet transform is a signal processing process, not a convolution operation, so no gradient derivation is required.

#### 2.2.3. Hybrid Domain Reconstruction Model

**Figure 4:**
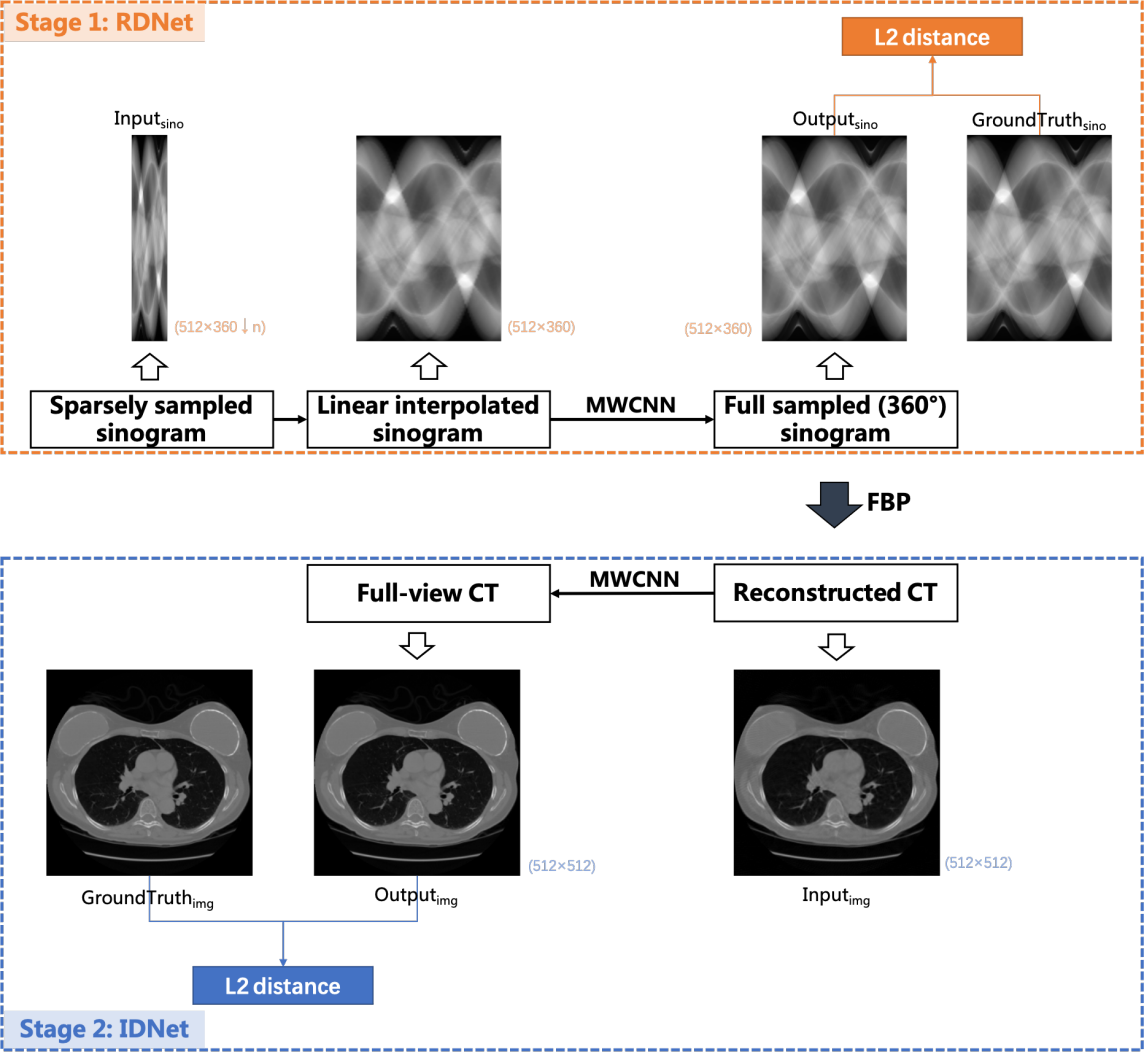
The hybrid domain reconstruction model for sparse-view CT.

A hybrid domain reconstruction model is proposed in this paper for sparsely sampled data, called Dual-domain Multi-level Wavelet Network (DuMWNet). In this model, the deep learning network MWCNN is applied to both radon domain and image domain. The workflow of sparsely sampled sinogram reconstruction is shown in Figure 4. It is a two-stage model that is characterized by setting the image reconstructed from radon domain predicted projections as the input of the image domain restoration network.

The first stage is a projection completion network in radon domain (RDNet). The input of the network is the sparsely sampled projection data, and the output is the estimated full-view projections. Because of the structural characteristics of the network, the sparsely sampled projections (sparse-view sinogram) will be firstly linearly interpolated to the same dimension as the full-view sinogram according to the angular interval. The MWCNN-based network helps to correct the error information caused by a linearly interpolated sinogram. The second stage is an image restoration network in image domain (IDNet), and the input is an image with a few artifacts reconstructed by FBP, which is a differentiable operator. The same network architecture is applied in an image domain to realize further image restoration.

The proposed hybrid-domain-based model is end-to-end learning in which RDNet and IDNet are trained in the same cycle because the domain transform operator is differentiable and its gradients can be computed and back propagation. The joint training loss function is expressed as

(6) $$L_{total}=L_{radon}+L_{image}={∥F_{radon}\left(x\right)-y∥}_{2}+{∥F_{image}\left(\mathrm{IR}\left(F_{radon}\left(x\right)\right)\right)-\mathrm{IR}\left(y\right)∥}_{2},$$

where $F_{radon}$ and $F_{imaage}$ represent networks of radon and image domains, IR represents the FBP operator, *x* is the sparsely sampled sinogram, and *y* is the full-sampled sinogram. $\left|\right|\cdot {\left|\right|}_{2}$ represents L2 loss.

## 3. Experimental Setting

### 3.1. Dataset

The LIDC-IDRI dataset is used in this paper, which was sponsored by the National Cancer Institute for the research of early cancer detection in high-risk populations. The dataset consists of chest image files and corresponding lesions labeled with diagnostic results. The image files are in DICOM format, which is the standard format of medical images. The image pixels have a size of 512 × 512 per slice, and there are some auxiliary elements such as image type, image time, and other information. In this study, we use 90 cases (16,826 slices) for training and another 10 patients (1870 slices) for testing.

### 3.2. Data Simulation

The LIDC-IDRI dataset only contains norm-dose CT images without projection data. A sinogram is used to display the projection data, where one row represents the projection data of a ray, and one column represents a view angle.

This paper uses parallel beam geometry to calculate the corresponding 360 degrees projections through computer simulation by using 360 detectors with the same image pixel size, and the sinogram with a size of 512 × 360 pixels is generated by the function radon. *iradon* is used for FBPs. In the sparse-view experiments, we uniformly subsampled the sinogram by factors of 3, 4, 6, and 12, and obtained 120, 90, 60, and even 30 views, respectively. The reconstruction process from radon domain to image domain adopts the traditional FBP algorithm.

### 3.3. Network Training

In addition to the classical algorithms FBP and SART-TV, several DL-based methods are applied as baselines to verify the advancement and effectiveness of the hybrid domain algorithm proposed in this paper. FBPConvNet [13], RED-CNN [14], DD-Net [22], and MWCNN [23] are image post-processing methods based on FBP reconstruction. A hybrid domain model [29] and HDNet [30] are dual domain methods based on FCN with one-level wavelet transform (WCNN) and U-Net, respectively. In addition, in order to verify that multi-level wavelet transform can eliminate artifacts better than a pooling layer, a hybrid domain reconstruction method based on U-Net with one-level wavelet transform was tested, which is also end-to-end learning.

RDNet and IDNet were optimized separately with the same hyper-parameters. We used the Adam algorithm with α = 0.0001, $\beta_{1}$ = 0.9, $\beta_{2}$ = 0.999 for optimizing and a mini-batch size of 4. The initial learning rate was set to be 0.0001 with a decay rate of 10% every 20 steps to avoid the impact of gradient fluctuations. We trained approximately 50 epochs. All DL models were built and trained in PyTorch.

### 3.4. Image Evaluation

In order to objectively evaluate the effectiveness of the image reconstruction method proposed in this paper, two image quality evaluation indexes, peak signal-noise ratio (PSNR), and structural similarity (SSIM) were used in this paper. PSNR is based on the error between corresponding pixels, so it is the unified expression of Mean Square Error (MSE). PSNR is expressed as:

(7) $$\mathrm{PSNR}=10log_{10}\left(\frac{{\mathrm{MAX}}_{Y}^{2}}{\mathrm{MSE}}\right),$$

where ${\mathrm{MAX}}_{Y}^{2}$ represents the maximum gray value of the standard image. Obviously, the smaller the MSE value, the larger the PSNR value, which indicates the better reconstruction effect.

SSIM can offset the shortcoming that MSE cannot measure the similarity of image structure. It measures image similarity from brightness, contrast, and structure, respectively, and is defined as:

(8) $$\mathrm{SSIM}\left(x,y\right)={\left[l\left(x,y\right)\right]}^{\alpha }\cdot {\left[c\left(x,y\right)\right]}^{\beta }\cdot {\left[s\left(x,y\right)\right]}^{\gamma },$$

where *l*, *c*, and *s* are brightness, contrast, and structure components, respectively. In practical application, α=β=γ=1, and the SSIM is expressed by

(9) $$\mathrm{SSIM}\left(x,y\right)=\frac{2\mu_{x}\mu_{y}+C_{1}}{\mu_{x}^{2}+\mu_{y}^{2}+C_{1}}\cdot \frac{2\delta_{x}\delta_{y}+C_{2}}{\delta_{x}^{2}+\delta_{y}^{2}+C_{2}}\cdot \frac{\delta_{xy}+C_{3}}{\delta_{x}\delta_{y}+C_{3}},$$

where $\mu_{x}$ and $\mu_{y}$ are the average pixel values of the images; $\delta_{x}$ and $\delta_{y}$ are the standard deviations of pixel values of the images; and $\delta_{xy}$ is the covariance of the two images. $C_{1}$, $C_{2}$, and $C_{3}$ are constants.

## 4. Results

We have conducted experiments on the sparse-view CT reconstruction task, and compared the experimental results with traditional and deep learning methods to verify the effectiveness of the algorithm proposed in this paper.

**Figure 5:**
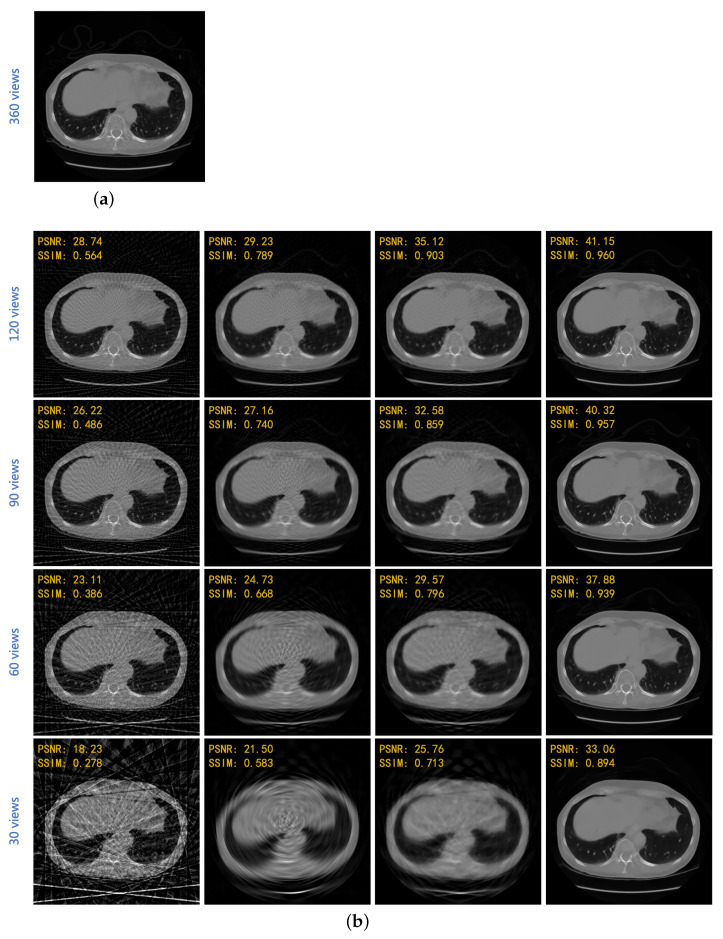
Reconstruction results of classical algorithms and the proposed one at different angular intervals. (**a**) Ground truth; (**b**) reconstruction results under four sparse coefficients. The first column is FBP, and the second one is FBP with a linearly interpolated sinogram. The third column is a TV-based SART algorithm, and the last one is the proposed method.

Three, four, six, and twelve angular intervals are employed for sparse-view experiments. Under different sampling conditions, the reconstruction results of the traditional methods and the proposed method are shown in Table 1 and Figure 5.

Figure 5b shows different reconstruction visions of an image from 1870 lung slices of the test sets at 3, 4, 6, and 12 angular intervals, respectively, corresponding to the raw sinogram of 512 × 120 pixels, 512 × 90 pixels, 512 × 60 pixels, and 512 × 30 pixels. To a certain extent, linear interpolation can suppress the artifacts, but the image details are lost. The SART algorithm based on total variation has a great improvement over FBP, but there are still subtle artifacts and the internal structure is reconstructed blurry. The deep learning method proposed in this paper has excellent reconstruction capability and can effectively compensate the artifacts of sparse-view CT. It is worth noting that the calculation time of SART-TV is about 1 min/slice, while the proposed method is about 1.8 s/slice, which is 33 times faster than the TV method.

**Figure 6:**
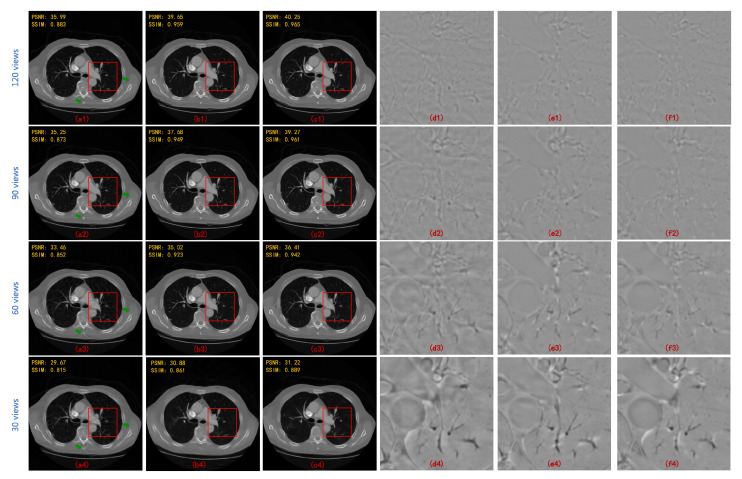
Reconstruction results of MWCNN applied to different domains at different angular intervals and the error maps of enlarged regions. (**a**) image domain; (**b**) radon domain; (**c**) hybrid domain; (**d**) error map of (**a**)—box-region; (**e**) error map of (**b**)—box-region; and (**f**) error map of (**c**)—box-region. The numbers (**1**–**4**) after the letter serial numbers represent the results of 120, 90, 60 and 30 projections, respectively.

In order to prove that the hybrid domain has a more prominent effect than a single domain, MWCNN is applied independently in radon domain, image domain, and hybrid domain. It should be noted that what is constructed in the radon domain is a sinogram inpainting network. The final reconstruction performance of processing in three different domains are shown in Figure 6a–c. The deep learning model in different domains can greatly suppress the streaking artifacts and image noise caused by sparse sampling. However, the image quality is slightly different. Specifically, the PSNR and SSIM values significantly increase by the hybrid domain reconstruction method. Error maps (compared with ground truth) of the region of interest in the images (i.e., the areas represented by the red box) are enlarged for better visualization, as shown in Figure 6d–f. It is obviously observed that the image reconstructed by the radon domain model can retain good spatial resolution, but there are still slight image artifacts. The image domain model has high quality reconstruction but low spatial resolution. The hybrid domain model provides the best reconstruction quality, which suppresses almost all image artifacts and contains high spatial resolution.

**Figure 7:**
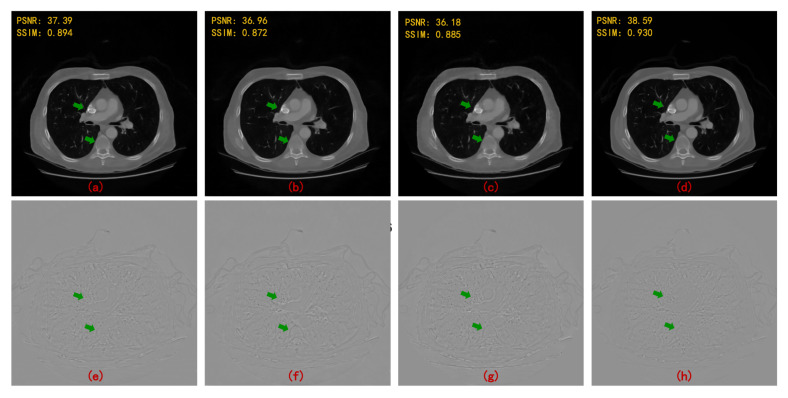
Reconstruction results and corresponding error maps of different post-processing methods at four angular intervals (90 views). (**a**) FBPConvNet; (**b**) RED-CNN; (**c**) DD-Net; (**d**) MWCNN; (**e**) error map of (**a**); (**f**) error map of (**b**); (**g**) error map of (**c**); and (**h**) error map of (**d**).

We compare the reconstruction performance of several image post-processing networks, including FBPConvNet, RED-CNN, DD-Net, and MWCNN to prove the effect of MWCNN on CT image denoising. Figure 7 shows the reconstruction results and their error maps compared with ground truth. All networks can eliminate streaking artifacts, but MWCNN can restore more image details. Compared with other DL networks, MWCNN achieves the best reconstruction indexes.

**Figure 8:**
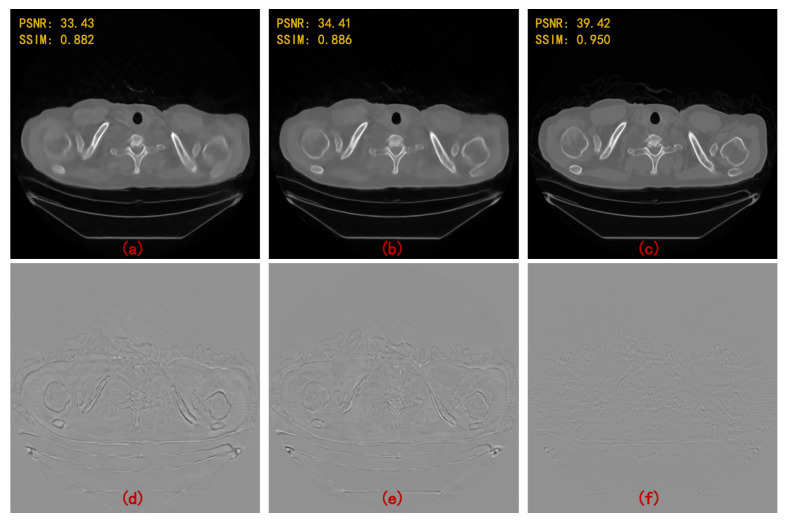
Reconstruction results and error maps of different hybrid domain methods at four angular intervals. (**a**) Dual-WCNN; (**b**) Dual-U-Net; (**c**) Dual-MWCNN; (**d**) error map of (**a**); (**e**) error map of (**b**); and (**f**) error map of (**c**).

Moreover, we compared the performance of MWCNN, single-level wavelet CNN (WCNN), and U-Net in the hybrid domain model, which further proves the effect of multi-level wavelet structure. MWCNN is a multi-resolution network with multi-level wavelet transform, while WCNN is a single-resolution network with one-level wavelet transform, and U-Net is a multi-resolution network without wavelet transform. Figure 8 shows the superior performance of MWCNN in sparse-view CT image reconstruction compared with U-Net and WCNN. It can be found that the internal and external areas restored by WCNN and U-Net are very blurry. The proposed method in this paper eliminates local small artifacts and restores the detailed image structure. It is worth noting that the network with wavelet transform can better preserve the detailed edge structure while compensating for directionality and eliminating artifacts.

**Figure 9:**
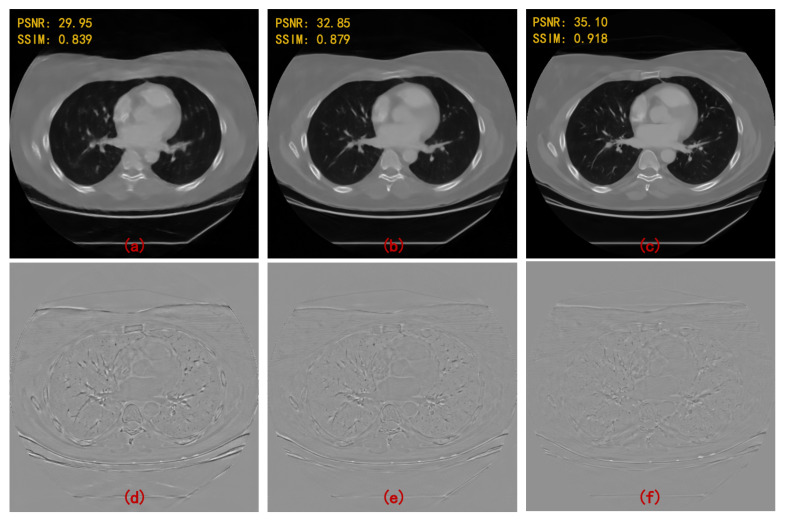
Reconstruction results and error maps of different wavelet-domain-based networks at 6 angular interval. (**a**) Dual-WCNN; (**b**) Dual-WUnet; (**c**) Dual-MWCNN; (**d**) error map of (**a**); (**e**) error map of (**b**); and (**f**) error map of (**c**).

Figure 9 shows the performance of CT reconstruction with single-resolution, multi-resolution network, and multi-level wavelet network in the wavelet domain. In the multi-resolution architecture, the receptive field is enlarged by the pooling operation, so it can better suppress the global artifact noise. The multi-level wavelet network uses wavelet transform instead of pooling operation to obtain stronger spatial feature extraction ability.

The reconstruction CT results of the deep learning methods above are shown in Table 2, which are the average PSNR and SSIM values of 1870 images of 10 patients. The hybrid domain method proposed in this paper achieves the maximum values both on PSNR and SSIM. HDNet can obtain higher SSIM but lower PSNR, indicating that the sinogram completion network model can enhance spatial structure restoration, but the noise suppression effect is not obvious. However, the hybrid domain model based on MWCNN has an improvement in both PSNR and SSIM, which proves that the multi-level wavelet network plays a great role in sinogram spatial context learning and global image artifacts suppression.

## 5. Discussion and Conclusions

Because of the global distribution of artifacts caused by sparsely sampled projections, the network structure of large receptive field can bring better learning performance. Meanwhile, the high directivity of artifacts makes learning in the wavelet domain superior to learning in the original image domain. Therefore, the MWCNN model is applied on sparse-view CT reconstruction, which is a network with a multi-level architecture and wavelet transform. In MWCNN, DWT is introduced to replace the pooling operation in the contracting subnet, and IWT is used to upsample the low-resolution feature maps to the high-resolution feature maps in the expanding subnet. Furthermore, a hybrid domain reconstruction method is proposed based on MWCNN, combining the sinogram restoration network in the radon domain and the image enhancement network in the image domain.

The proposed method achieves far better results than traditional algorithms, both on PSNR and SSIM. At the same time, the experimental comparison shows that the multi-level wavelet transform model is more suitable for sparse-view CT reconstruction than the single-level wavelet transform because the expanding receptive field in MWCNN can easily capture the streaking artifacts with global distribution. The introduction of wavelet transform also makes the removal of artifacts more natural than the traditional U-Net. The reconstruction results of the network applied on different domains verify the validity of the multi-domain joint model.

## Figures and Tables

**Table 1:** Average PSNR and SSIM results of the traditional and proposed methods at different angular intervals.

	FBP	Linear + FBP	SART-TV	Proposed
120 views	28.725	30.241	34.405	**41.049**
(3-degree)	0.558	0.802	0.903	**0.958**
90 views	26.702	28.475	32.190	**40.204**
(4-degree)	0.483	0.765	0.862	**0.956**
60 views	23.259	26.279	30.085	**37.718**
(6-degree)	0.391	0.698	0.810	**0.938**
30 views	19.496	23.399	26.711	**33.100**
(12-degree)	0.280	0.624	0.726	**0.891**

**Table 2:** Average PSNR and SSIM results for various deep learning reconstruction methods.

	90 Views		60 Views	
	PSNR	SSIM	PSNR	SSIM
FBPConvNet [13]	37.611	0.921	35.578	0.896
RED-CNN [14]	37.209	0.902	34.528	0.859
DD-Net [22]	36.380	0.912	34.424	0.892
MWCNN [23]	38.664	0.943	36.531	0.921
HDNet (WCNN-based) [29]	34.506	0.900	32.259	0.869
HDNet (Unet-based) [30]	35.459	0.903	33.377	0.886
HDNet (WUnet-based)	36.750	0.924	35.132	0.904
**HDNet (MWCNN-based)**	**40.204**	**0.956**	**37.718**	**0.938**

## Data Availability

Publicly available datasets were analyzed in this study. This data can be found here: https://wiki.cancerimagingarchive.net/display/Public/LIDC-IDRI (accessed on 24 January 2021).
